# Optimization and Modeling of Various Fermentation Parameters Influencing Liamocin Production by *Aureobasidium pullulans* NBRC 100716 Strain

**DOI:** 10.1021/acsomega.5c00804

**Published:** 2025-08-06

**Authors:** Aslı Deniz Pehlivan, Melek Tijen Bozdemir, Zekiye Yesim Ozbas

**Affiliations:** † Faculty of Engineering, Department of Food Engineering, Hacettepe University, Beytepe, 06800 Ankara, Turkey; ‡ Faculty of Engineering, Department of Chemical Engineering, Hacettepe University, Beytepe, 06800 Ankara, Turkey

## Abstract

Liamocins are unique extracellular glycolipid biosurfactants produced by certain species of *Aureobasidium pullulans* with potential applications in agriculture, food, medicine, and pharmaceutical industries due to their surface-active properties and antimicrobial activity. In this study, for the first time, optimization of various fermentation parameters for liamocin production by *A. pullulans* NBRC 100716 strain was carried out using the response surface methodology (RSM). By conducting numeric optimization, the optimum conditions were determined as follows: initial substrate (fructose) concentration (*X*1) of 93.47 g/L, initial pH (*X*2) of 4.92, and temperature (*X*3) of 27.2 °C. Under optimum conditions, the maximum liamocin concentration (*Y*1), microorganism-specific growth rate (*Y*2), and maximum specific liamocin production rate (*Y*3) were found to be 3.56 g/L, 0.0670 h^–1^, and 0.00450 [g liamocin/(g mo·h)], respectively. Variations of the responses with the independent variables were defined by a quadratic model. According to the analysis of variance (ANOVA) results, the model terms in the study showed high significance (*p* < 0.05) for all response variables, while the lack of fit was not significant for any of the responses. The coefficient of determination for liamocin production (0.829) was close to 1.0, confirming the significance of the model. Matrix-assisted laser desorption ionization time-of-flight mass spectrometry (MALDI-TOF-MS) determined that the liamocin structure contained all liamocin types referred to as A1, B1, A2, and B2. By the gas chromatography-flame ionization detection (GC-FID) method, it was determined that 61.12% of the fatty acid composition consisted of saturated and 38.87% consisted of unsaturated fatty acids (UFA). Furthermore, Fourier transform infrared spectrometry (FT-IR) spectral attributes were used for the first time to confirm the structure of liamocin, providing valuable insights. This study, in which some fermentation parameters were optimized, showed that the *A. pullulans* NBRC 100716 strain is promising for industrial-scale liamocin production in the future.

## Graphical abstract

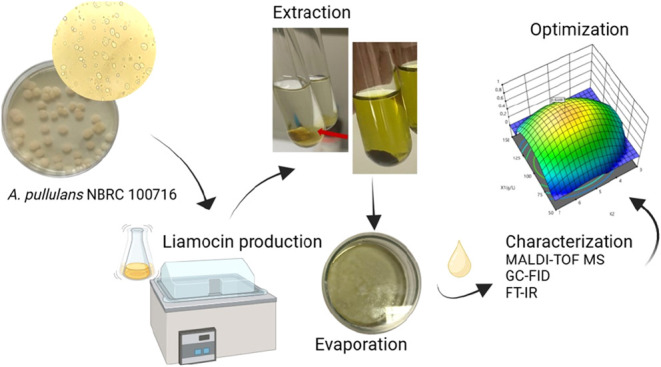

## 1 Introduction

Liamocins are extracellular heavy oils in a polyol lipid structure produced by rare *Aureobasidium* spp. such as *A. pullulans*. Typical chemical structures of liamocins have been reported to consist of a single partially O-acylated mannitol or arabitol headgroup with polyester tails containing three or four 3,5-dihydroxydecanoic ester groups, which may be acetylated.^1^ It has been determined that the polyol headgroup can vary depending on the microorganism strain, culture medium, and type of polyol used. It has also been reported that depending on the type of polyol used in the fermentation medium, the headgroup may consist of different polyols such as mannitol, arabitol, glycerol, or xylitol.^2,3^

There are four types of liamocin that have been structurally characterized so far: liamocin A1, A2, B1, and B2. It has been reported that liamocins A1 and A2 contain three 3,5-dihydroxydecanoic ester groups, while liamocins B1 and B2 contain four 3,5-dihydroxydecanoic ester groups. While liamocin A1 and B1 are not acetylated, A2 and B2 each contain a single 3′-O-acetyl group.^2^

There are still a few studies in the literature about liamocin molecules in the polyol lipid structure, which have high potential to be used in food, agriculture, medicine, cosmetic, and pharmaceutical industries.^3,4^

It has been reported that liamocins act as biosurfactants that are able to reduce surface tension like commercially used surfactants.^5^ Liamocin molecules have been shown to inhibit the proliferation of some cancer cell lines and are also potential selective antimicrobial molecules.^6^ Liamocins produced by various *A. pullulans* strains have been reported to have specific antimicrobial activity against some *Streptococcus* species.^7^ Therefore, it has also been reported that liamocin has the potential to be a new antimicrobial agent and can be used as a target-specific antimicrobial agent against pathogenic strains with antibiotic resistance.^8^

Liamocin is defined as an important biomolecule that will have the potential for use in many industrial areas in the future. However, there are some problems to be overcome, especially in large-scale production. Low productivity, costly raw materials, and expensive downstream processing and purification have been reported to cause high cost in liamocin production.^7^ For these reasons, it is important to optimize liamocin production with various strains.

Liamocin production by *A. pullulans* strains may vary depending on the type of carbon source used in the fermentation medium. In previous studies, the most frequently used carbon source in liamocin production was reported as sucrose.^3,6^ In a study conducted by Price et al.,^2^ the effects of various sugar and polyol carbon sources, including sucrose, lactose, and fructose, on liamocin production were investigated. The highest concentration of liamocin (4.4 g/L) was determined in the medium containing sucrose as the carbon source, followed by the liamocin concentration in the medium using fructose (4.1 g/L). In our previous study examining the effects of different carbon sources on liamocin production and growth of *A. pullulans* NBRC 100716 strain, it was found that the highest liamocin production concentration for this strain was obtained in fermentation experiments using fructose.^9^

It has also been stated that various parameters such as pH, temperature, aeration, incubation time, and inoculum ratio may affect liamocin production of strains. In the studies conducted, it is reported that the most commonly used pH values in fermentation media for liamocin production vary between 5.5 and 7.0.^3^ The temperature range used in researches for liamocin production is reported as 25–30 °C. Depending on the strain, 25 or 28 °C is widely preferred.^6,10^ In addition, the inoculum ratio used in liamocin fermentation varies between 8 and 10%.^3^

There are few studies in the literature on the optimization of various fermentation parameters for liamocin production.^11,12^ A study was conducted by Leathers et al.^11^ to optimize the composition of the fermentation medium for liamocin production in a batch system. In the mentioned study, a Plackett–Burman design was used. It was stated that the concentration of liamocin (22 g/L) produced by *A. pullulans* NRRL 50384 strain increased to approximately double compared to the standard medium. In another study performed in a bioreactor using *A. pullulans* NRRL 62042 strain, an experimental design approach including a two-level factorial design followed by a central composite design was used to develop a minimal medium for liamocin production.^12^

The major aim of this study was to optimize some fermentation parameters in order to improve liamocin production of the tested *A. pullulans* strain. For this purpose, the effects of initial substrate (carbon) concentration, initial pH, and temperature on liamocin production in the batch fermentation system were investigated for the first time using the response surface methodology (RSM). In addition, to our knowledge, this is the first study to determine that the produced liamocin is a biosurfactant with a glycolipid structure, seen by Fourier transform infrared spectrometry (FT-IR).

## 2 Materials and Methods

### 2.1 *A. pullulans* Strain

In this research *A. pullulans* NBRC 100716, which has been proven to be a liamocin producer in our previous studies, was used.^9^ This strain was kindly obtained from the Biological Resource Center, NITE (NBRC), Japan, in a lyophilized form. The culture was maintained on a Yeast Extract Malt Extract (YM) agar slant at 4 °C and was subcultured every 2 weeks.

### 2.2 Media and Preparation of Inoculum

In this study, *A. pullulans* NBRC 100716 strain was activated in Yeast Extract Malt Extract (YM) broth medium containing (g/L) yeast extract, 3; malt extract, 3; peptone, 5; and glucose, 10. Cultures were kept on YM agar medium at 4 °C until use.

In all of the experiments, the inocula were prepared by incubation at 28 °C for 48 h in a culture medium containing (g/L) sucrose, 50; peptone, 0.6; yeast extract, 0.4; K_2_HPO_4_, 5; MgSO_4_.7H_2_O, 0.4; NaCl, 1.^6^ The experiments were carried out using a fermentation medium containing fructose as the substrate. Other components of the fermentation medium were kept the same as in the culture medium. In our previous studies, it was found that the *A. pullulans* NBRC 100716 strain is a fructophilic strain and the substrate that best supports liamocin production of this strain is fructose.^9^

All media were sterilized in an autoclave at 121 °C for 15 min. The pH of the fermentation medium was measured using a Hanna HI 221 model (Italy) pH meter, and the initial pH of the medium was adjusted to 6.5 using 1 mol/L HCl (Sigma-Aldrich).

For fermentation experiments, the initial inoculum concentration (CFU/mL) of the tested strain was determined by a cultural method by using YM agar medium.

### 2.3 Experimental Setup for Liamocin Fermentation

Fermentation experiments were carried out in a batch system using a water bath (Nüve ST-402, Turkey) with a controllable shaking speed and temperature.

For fermentation, the inoculum prepared as previously described was inoculated at 10% (v/v) into 100 mL of fermentation medium in a 300 mL cotton-plugged flask, and incubation was carried out at 28 °C in a dark environment at a constant shaking speed of 100 strokes per minute. During the experiments, 10 mL samples were taken from the fermentation media approximately every 24 h, and liamocin, biomass, and substrate concentrations were determined.

### 2.4 Determination of Liamocin

In this study, the method suggested by Manitchotpisit et al.^6^ was slightly modified and used to determine liamocin produced in the fermentation medium. In the analysis, first, a 10 mL sample taken from the fermentation medium was centrifuged at 5000 rpm for 20 min (Nüve, NF 615, Turkey) and the supernatant was removed. Then, 3 mL of distilled water was added to the remaining precipitate (biomass) and the mixture washed by centrifuging again under the same conditions.

Next, the supernatant was separated, and 3 mL of 99.5% methyl ethyl ketone (Birpa, Turkey) was added to the pellet and the mixture was vortexed (Isolab, Turkey) vigorously. The mixture was then placed in a fume hood system and kept under an air stream for 90 min for solvent extraction.

After extraction, the sample was vortexed vigorously and centrifuged again at 5000 rpm for 20 min. The supernatant obtained after centrifugation was carefully poured into sterile and previously tared Petri dishes, and the solvent was allowed to evaporate. After the solvent was evaporated from the mixture, the liamocin samples were weighed and the liamocin concentration (g/L) was calculated.

### 2.5 Determination of Biomass Concentration

In this research, biomass concentrations in samples taken from fermentation media were determined by dry cell weight analysis.^13^ For this purpose, the pellet remaining after liamocin extraction was dried in an oven (Memmert, Germany) set at 80 °C until a constant weight was reached. The sample was then weighed, and the biomass concentration (g/L) in the fermentation medium was calculated.

### 2.6 Determination of Substrate Concentration

During the experiments, concentrations of fructose used as substrate in the fermentation media were determined by the dinitrosalicylic acid (DNS) method.^14^ Calibration curve was drawn for the sugar to be analyzed, and measurements were made using a spectrophotometer (Boeco S-30, Germany) at a wavelength of 540 nm.

### 2.7 Determining Kinetic Characteristics of Microbial Growth and Liamocin Production

In this study, the maximum liamocin and maximum biomass concentrations (Xm), specific microbial growth rates (μ), maximum specific liamocin production rates (qp), and substrate consumption rates (*r_S_*) were calculated in the fermentation experiments. The specific microbial growth rate (μ) for the exponential growth phase was calculated from the semilogarithmic plot of the dry biomass (*X*) data versus time (*t*) (eq 1)^15^

1 $$\frac{dX}{dt}=\mu X$$

Using the changes in liamocin and dry biomass concentrations with time, specific liamocin production rates (qp) were calculated from^16,17^

2 $$\mathrm{qp}=\frac{1}{X}\;\frac{dP}{dt}$$

In this equation, d*P*/d*t* is the product formation rate and *X* is the microorganism concentration at the time the product (liamocin) formation rate is calculated.

The substrate consumption rates (*r_S_*) (eq 3) were also calculated by finding the slope values at different times from the curves in graphs showing the changes in substrate concentrations’ changes with time

3 $$r_{S}=-\frac{dS}{dt}=-\frac{dS}{dX}\;\frac{dX}{dt}=-\frac{dS}{dX}\mu X$$

where *S* is the substrate concentration in g of substrate/L.

Calculations of kinetic constants and rates were performed by using the Origin Pro 8.5 (Origin lab Corporation, Northampton) program package.

### 2.8 Experimental Design for Liamocin Production

Response surface methodology (RSM) was used to optimize some fermentation parameters in the production of liamocin by the tested *A. pullulans* strain. This optimization was carried out using the Central Composite Rotatable Design (CCRD) in the RSM.

For this purpose, the statistical software Design Expert trial version 12 (Stat-Ease Inc., Minneapolis, MN) was used, and the optimal combination of variables and response patterns was determined. The experimental design was carried out at five different levels coded as −1.68, −1, 0, +1, and +1.68 and consisted of a total of 20 experimental points with 14 different combinations, with six repetitions at the central point. The star point (α) value, which expresses the process range in the design, is calculated according to the following equation

4 $$\alpha ={{[2}^{k}]}^{1/4}$$

The number of experiments in the design is calculated by the program according to the equation below

5 $$n=2^{k}+2k+n_{0}$$

In this study, the independent variables investigated for optimization are initial fructose concentration of the fermentation medium (50–150 g/L), initial pH (3–7), and temperature (24–32 °C) (Table 1).

**Table 1:** Independent Variables and Their Levels Used for the Experimental Design

		levels of actual values of coded factors				
independent variables	symbols	–1.68	–1	0	+1	+1.68
initial fructose concentration (g/L)	*X*1	50.00	70.27	100.00	129.73	150.00
pH	*X*2	3.0	3.8	5.0	6.2	7.0
temperature (°C)	*X*3	24.00	25.6	28.0	30.4	32.0

The selection of the minimum initial fructose concentration in the fermentation medium was determined based on the concentration of fructose used in the study of Manitchotpisit et al.^6^ Since there was no study examining the effect of different initial fructose concentrations on liamocin production, the selection was made by considering the researches that investigated the maximum carbon source concentrations used for the production of various metabolites by *A. pullulans*.^18,19^

The minimum and maximum values for the pH range were determined also based on literature data. The minimum pH was selected as 3.0, which is close to the lowest pH value (3.5) reported for liamocin production in the literature.^20^ The maximum pH was chosen as 7.0, considering that values changing between 6.0 and 6.5 were generally used in the previous studies.^6,11^

In the literature, a narrow temperature range (25–30 °C) has been used for liamocin production. To the best of our knowledge, no study has specifically focused on temperature optimization. However, the highest liamocin yield has been obtained at 25 °C.^10^ Based on these data, the minimum temperature in our study was set at 24 °C, while the maximum temperature was determined as 32 °C, considering in another research in the literature reporting as the optimum temperature for *A. pullulans* cell growth.^21^

In this study, each independent variable was studied at five coded levels for three responses (Table 1). As responses (dependent variables), maximum liamocin concentration, *Y*1 (g/L), microorganism-specific growth rate (μ), *Y*2 (h^–1^), and maximum liamocin production rate (qp), *Y*3 (g liamocin produced per g microorganism per hour [g liamocin/(g microorganism·h)]) [g liamocin/(g mo·h)] values were selected.

In this part of the research, all experiments in the experimental design plan were carried out under the operating conditions given in the plan for each experiment. During the experiments, culture samples were taken from the fermentation medium at certain time intervals, and analyses were performed to determine the concentrations of liamocin, substrate, and biomass. Then, graphs presenting the calculated liamocin, substrate, and biomass concentrations versus time for each experiment were prepared using the Origin Pro 8 (Origin Lab Corporation, Northampton) program.

### 2.9 Modeling of Dependent Variables with the Response Surface Method

Quadratic model equations showing the effects of the independent variables selected in the study on the dependent variables were derived using multiple regression analysis by using the trial version of Design Expert 12 (Stat-Ease Inc., Minneapolis) software. To analyze the experimental data, the second-order polynomial equation (eq 6), given below, was used

6 $$y=\beta_{0}+\sum_{i=1}^{k}\beta_{i}X_{i}+\sum_{i=1}^{k}\beta_{\mathrm{ii}}X_{i}^{2}+\sum_{i=1}^{k}\sum_{j=1}^{k}\beta_{\mathrm{ij}}X_{\mathrm{ij}}+\varepsilon$$

where *y* is the response, β_0_ is the constant coefficient, β*_i_*, β*_ii_*, and β*_ij_* are linear, quadratic, and interaction regression coefficients, ε is the error of the model, and *X_i_* and *X_j_* are independent variables.^22,23^

In addition, the statistical significance of the model equations and interactions between process variables and responses were determined using *F*-test by performing an analysis of variance (ANOVA). The acceptabilities of the models were evaluated mainly based on the significance of the model (*p* < 0.05), insignificant lack of fit (*p* > 0.05), and coefficient of determination (*R* ^2^) value.

In addition, the optimal levels of the variables obtained as a result of statistical analysis were also examined using the response surface and 3D surface graph plots drawn using the Design Expert 12 (Stat-Ease Inc., Minneapolis) program.

### 2.10 Optimization and Validation of the Experimental Model

Optimization was performed by numerical analysis based on the desirability function by using the Design Expert program. The optimum levels of the variables were obtained by numerical analysis using the software. Numerical optimization finds a point that maximizes the desirability function.^24^

One of the most important goals of this study was to produce liamocin in a high concentration. Therefore, only the maximum liamocin concentration (*Y*1) target was selected as the “maximum”. In the study, no target was specified for the microorganism-specific growth rate (*Y*2), and the maximum specific liamocin production rate (*Y*3) was selected as “in range”. Furthermore, the objectives were also determined for independent variables. Initial fructose concentration was used as “in range” between 50 and 150 g/L; initial pH was “in range” between 3 and 7, and temperature was “in range” between 24 and 32 °C.

After selecting the minimum and maximum values of the independent (Table 1) and dependent variables, numerical optimization was performed with the Design Expert program, and different solutions suggested by the software were obtained. The desirability function values of the solutions were also given by the software. Then, a solution with the highest desirability function value (close to 1.0) was selected and an experiment was performed in parallel with the independent variable values given in the solution. These experiments were performed in a shaking water bath, as previously described (see Section 2.3). In the optimization experiment, 10 mL culture samples were taken from the fermentation media approximately every 24 h to determine the concentrations of liamocin, biomass, and substrate in the medium. Then, graphs showing the change of the values calculated from the obtained data with time were drawn. Using these graphs, maximum liamocin concentration (*Y*1), microorganism-specific growth rate (*Y*2), maximum specific liamocin production rate (*Y*3), and substrate consumption rate (*r_S_*) values were calculated for the experiments carried out under optimum conditions. After the experimental values were obtained under optimum conditions, the confirmation menu of the Design Expert program was used to verify the developed model. The validity of the obtained responses for the optimum conditions was evaluated by taking into account the maximum and minimum values predicted at the 95% confidence interval.

### 2.11 Characterization of Liamocin Samples

#### 2.11.1 Nile Red Staining

Nile red is a fluorescent lipophilic dye used for the detection and quantification of lipids in algae, yeasts, and filamentous fungi.^25^ In this study, Nile Red staining was used to observe liamocin (heavy oil) produced by *A. pullulans* NBRC 100716. The staining process was applied to *A. pullulans* culture grown in liamocin fermentation medium (see Section 2.2) at 28 °C for 168 h. Nile Red dye (Sigma-Aldrich), dissolved at a concentration of 0.05 mg in 100 mL of dimethyl sulfoxide (DMSO; Sigma-Aldrich) was applied to slides prepared from the culture for 10 min. Stained slides were examined using a fluorescence microscope (Nikon Eclipse TS100) with a 40× objective^26^ and images were recorded using NIS-Elements software.

#### 2.11.2 Oil Red O Staining

Oil red O is a fat-soluble dye used to stain neutral lipids, cholesteryl esters, and lipoproteins. Oil red staining is a technique used to visualize lipids in tissues or cells. Due to its cellular permeability, it is widely used for intracellular lipid staining and tissue staining. During staining, Oil red O is transferred from the dye solution to the lipid, resulting in the lipid droplets being stained red.^27^

In this study, the Oil red O staining method was used to observe the liamocin produced by the tested *A. pullulans* strain. For this purpose, first the dye solution was prepared by dissolving 0.5 g of Oil red O (Merck, Germany) in 100 mL of isopropyl alcohol.^28^ The strain was grown in the culture medium (see Section 2.2) at 28 °C for 48 h. Slides prepared from these cultures were stained with the dye solution and kept at room temperature for 5 min. The stained cultures were then examined under a light microscope (Olympus BX50, Japan) using immersion oil at 100× magnification, and the oil formations inside and outside the cells were evaluated.

#### 2.11.3 Structural Characterization

In this study, matrix-assisted laser desorption ionization time-of-flight mass spectrometry (MALDI-TOF-MS) analysis was used to determine the structural characterization of the liamocin sample produced by *A. pullulans* NBRC 100716. The dried droplet method was used to prepare the liamocin sample for the analysis.^29^ In the analysis, liamocin sample dissolved in 1 mL of acetonitrile was mixed with an equal volume of matrix solution (0.1% formic acid in acetonitriledihydroxybenzoic acid (DHBA) solution (50:50, v/v)), and 0.5 μL of sample was dropped onto the target steel plate and allowed to dry at room temperature. Analysis was carried out on a MALDI-TOF-MS instrument (Ultraflextreme MALDI-TOF, Bruker Daltonics). 337 nm UV laser was operated at an accelerating voltage of 20 kV. The spectrum was collected in reflectron positive ion mode with 10,000 laser shots and in the *m*/*z* range of 450–1350 Da. Spectra were obtained using Bruker flexAnalysis Software (Bruker Daltonics, Germany).

#### 2.11.4 Gas Chromatography-Flame Ionization Detection (GC-FID)

In this study, the fatty acid profile of the liamocin sample produced by the *A. pullulans* NBRC 100716 strain was determined using the GC-FID method. For this purpose, the method suggested by Wang et al.^30^ was used. In the method, first, the methyl esters of the sample were formed with methanolic KOH and analyzed with a GC-FID (Thermo Scientific) equipped with a column of RTX-2230 (0.25 mm × 30 m, 0.20 μm), and helium was used as carrier gas at a flow rate of 1 mL/min. The GC oven was set as follows: 70 °C (2 min hold), ramped to 120 °C at 4 °C/min, 180 °C at 22 °C/min (3 min hold), 200 °C at 4 °C/min (3 min hold), and finally ramped to 230 °C at 7 °C (5.21 min hold). As a standard, Supelco 37 Component FAME Mix (Sigma-Aldrich) was used. The identification of fatty acids was performed by comparing with the relative retention time of the FAME peaks from the sample with standards, and the percentage of fatty acid composition was then calculated based on the normalization of detected peak areas.

#### 2.11.5 Fourier Transform Infrared Spectrometry (FT-IR)

The molecular structure of the liamocin sample produced by *A. pullulans* NBRC 100716 was also determined by using an FT-IR spectrometer (Thermo Fisher Scientific Smart iTX), at wavelengths from 500 to 4000 cm^–1^, with 32 scans at a resolution of 4.0 cm^–1^. With this equipment, the liamocin sample was analyzed directly without any sample preparation.

## 3 Results and Discussion

### 3.1 Experimental Design for Liamocin Production

The central composite rotatable design matrix (CCRD) with the calculated values of maximum liamocin concentration (*Y*1), microorganism-specific growth rate (*Y*2), and maximum specific liamocin production rate (*Y*3) are given in Table 2.

**Table 2:** CCRD Matrix Indicating the Actual Values of Independent Variables and the Calculated Values of Responses^a^

	independent variables			responses		
run	*X*1 (g/L)	*X*2	*X*3 (°C)	*Y*1 (g/L)	*Y*2 (h^–1^)	*Y*3 [g liamocin/(g mo·h)]
1	150.00	5.0	28.0	0.82	0.040	0.0012
2	129.73	3.8	30.4	0.42	0.058	0.0020
3	129.73	6.2	25.6	0.92	0.035	0.0020
4	100.00	5.0	28.0	4.31	0.078	0.0063
5	70.27	3.8	30.4	0.47	0.053	0.0020
6	100.00	5.0	28.0	5.30	0.062	0.0065
7	129.73	6.2	30.4	0.44	0.060	0.0011
8	100.00	5.0	28.0	6.10	0.076	0.0063
9	50.00	5.0	28.0	1.12	0.054	0.0017
10	100.00	5.0	24.0	0.60	0.056	0.0016
11	129.73	3.8	25.6	1.16	0.029	0.0015
12	100.00	5.0	32.0	1.50	0.044	0.0029
13	70.27	6.2	30.4	0.43	0.078	0.0017
14	100.00	5.0	28.0	4.05	0.078	0.0043
15	100.00	7.0	28.0	5.63	0.053	0.0063
16	100.00	5.0	28.0	5.62	0.083	0.0065
17	100.00	5.0	28.0	4.71	0.089	0.0057
18	100.00	3.0	28.0	0.61	0.033	0.0016
19	70.27	3.8	25.6	2.25	0.028	0.0032
20	70.27	6.2	25.6	1.12	0.053	0.0020

a *X*1: initial fructose concentration (g/L), *X*2: initial pH, *X*3: temperature (°C), *Y*1: maximum liamocin concentration (g/L), *Y*2: microorganism-specific growth rate (h^–1^), *Y*3: maximum specific liamocin production rate [g liamocin/(g mo·h)].

The experiments were carried out as 6 parallel runs performed under the same conditions at the central point, and 14 different combination experiments at the star and factorial points, for a total of 20 experiments. Experiments were performed at different initial fructose concentrations (50–150 g/L), initial pH (3–7), and temperatures (24–32 °C) (see Section 2.10).

As seen from the data in Table 2, the maximum liamocin concentrations (*Y*1) ranged between 0.42 and 6.10 g/L. In run number 8, where the initial fructose concentration was 100 g/L, the initial pH was 5.0, and the temperature was 28.0 °C, the maximum liamocin concentration (6.10 g/L) was achieved. The minimum liamocin concentration was obtained as 0.42 g/L in the run where the initial fructose concentration was 129.73 g/L, the initial pH was 3.8, and the temperature was 30.4 °C (at run 2). In the 20 runs performed (Table 2), specific growth rates (*Y*2) ranged from 0.028 to 0.089 h^–1^ and the maximum specific liamocin production rates (*Y*3) ranged from 0.001 to 0.0065 [g liamocin/(g mo·h)].

The second-order polynomial model equation describing the change of the liamocin concentration (*Y*1) by the independent variables (*X*1, initial fructose concentration of the fermentation medium; *X*2, initial pH of the fermentation medium; *X*3, temperature) is given below

7 $$Y1=+5.03-0.1783X1+0.4725X2-0.2033X3+0.1938X1X2+0.2313X1X3+0.2438X2X3-1.54{\left(X1\right)}^{2}-0.7833{\left(X2\right)}^{2}-1.52{\left(X3\right)}^{2}$$

The model was found to be significant (*p* < 0.05) with insignificant lack of fit and coefficient of determination (*R* ^2^) value of 0.8292.

The ANOVA table for the quadratic model of *Y*1 is shown in Table 3.

**Table 3:** ANOVA Results for the Model Predicted for Maximum Liamocin Concentration (*Y*1)

source	df	mean squares	*F*-value	*p*-value	remarks
model	9	7.82	4.71^a^	0.0118	significant
*X*1	1	0.4340	0.2617	0.6201	not significant
*X*2	1	3.05	1.84	0.2050	not significant
*X*3	1	0.5644	0.3403	0.5726	not significant
*X*1*X*2	1	0.3003	0.1811	0.6795	not significant
*X*1*X*3	1	0.4278	0.2579	0.6226	not significant
*X*2*X*3	1	0.4753	0.2866	0.6041	not significant
*X*1^2^	1	34.33	20.70^a^	0.0011	significant
*X*2^2^	1	8.84	5.33^a^	0.0436	significant
*X*3^2^	1	33.08	19.95^a^	0.0012	significant
lack of fit	5	2.69	4.27	0.0685	not significant
pure error	5	0.6291			
cor total	19				

a *p* < 0.05.

*F*-value of the model (4.71) implies the model is significant (Table 3). There is only a 1.18% chance that an *F*-value this large could occur due to noise. The “lack of fit *F*-value” of 4.27 implies there is a 6.85% chance that a lack of fit *F*-value this large could occur due to noise.

Lack of fit is an important aspect of ANOVA. It shows the functional relationship of independent variables with dependent responses. A minor lack of fit is good and indicates the fit of the current model.^31^ In this research, the lack of fit in all models for liamocin production was found to be insignificant (*p* > 0.05).

The model’s statistical suitability and significance were assessed using *p*-value and Fishers test value (*F*-value). The *p*-value and *F*-value were used to statistically evaluate the overall significance of the regression model and the fit of model terms and to evaluate specific regression terms. The statistical significance of the created model is shown by a lower *p*-value and higher *F*-value.^31^ The model terms of the conducted study showed high significance with *p* < 0.05 for all response variables. The good quality of model is indicated by the value of the regression coefficient. The *R* ^2^ value is very close to 1, indicating good agreement between the predicted and experimental values.

According to the ANOVA results (Table 3), the model showing the effects of the independent variables examined on the maximum liamocin concentration was found to be significant (*p* < 0.05), while the lack of fit was found to be insignificant (*p* > 0.05). In addition, while the binary interactions of the independent variables examined (*X*1*X*2, *X*1*X*3, and *X*2*X*3) were found to be insignificant, the effects of the quadratic effects of each variable on the maximum liamocin concentration were found to be significant. 3D surface graph plots were used to examine the impact of independent factors and their interactions with desired responses. By maintaining three variables at their optimal levels and adjusting the other two within their experimental ranges, 3D surface plots were generated.

Figure 1 presents the response surface and contour plots showing the effects of the initial fructose concentration or initial pH of the fermentation medium and temperature on the maximum liamocin concentration.

**Figure 1:**
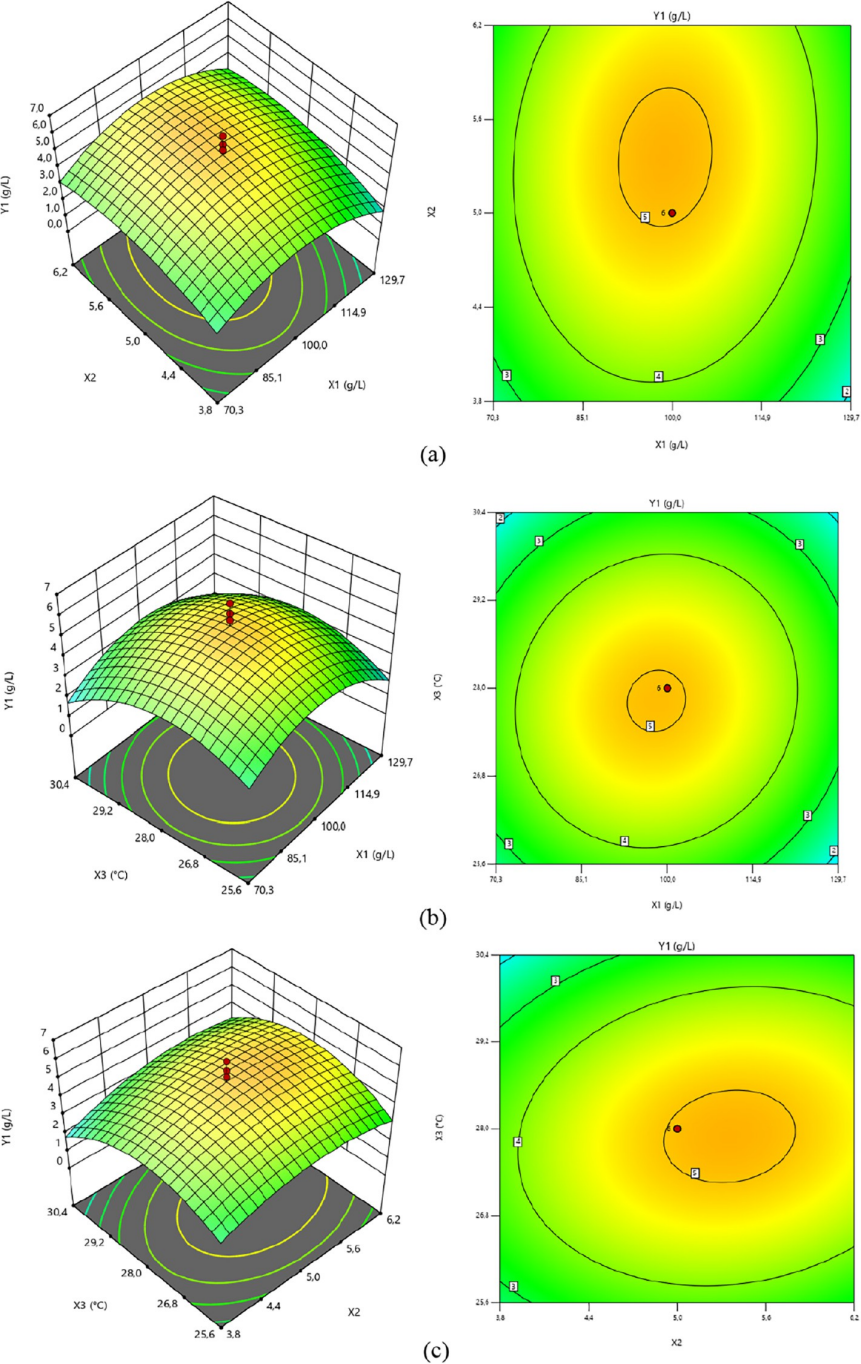
Response surface and contour graphs of maximum liamocin concentration (*Y*1) as a function of (a) initial fructose concentration (*X*1) and initial pH (*X*2), (b) initial fructose concentration (*X*1) and temperature (*X*3), and (c) initial pH (*X*2) and temperature (*X*3).

In this study, independent variable values, temperature, *X*3, in Figure 1a; initial pH of the fermentation medium, *X*2, in Figure 1b; and initial fructose concentration of the fermentation medium, *X*1, in Figure 1c, were kept constant with their corresponding values, same as that in the center point.

As can be seen from the response surface plot in Figure 1a, when the initial fructose concentration in the fermentation medium is high (in the range 120–129 g/L) and the initial pH is in the range 3.8–4.2, the maximum liamocin concentration is below 2.60 g/L.

While the initial fructose concentration and pH value were in the ranges of 90–110 and 4.8–5.7 g/L, respectively, the liamocin concentration was close to the maximum and varied between 4.90 and 5.10 g/L. In addition, the highest liamocin concentration (5.10 g/L) was obtained when the initial fructose concentration was 100 g/L and pH was 5.30. As can be seen from the contour plot in Figure 1a, the liamocin concentration was high (above 5 g/L) when the initial fructose concentration was between 90 and 106 g/L and the pH was between 4.9 and 5.8.

Response surface and contour plots showing the effects of the fermentation medium initial fructose concentration (*X*1) and temperature (*X*3) on the maximum liamocin concentration are presented in Figure 1b. According to Figure 1b, high liamocin concentrations (4.80–5.02 g/L) were reached at initial fructose concentrations in the range of 90–105 g/L, and temperature between 25 and 26 °C. It is clear from Figure 1b that the maximum liamocin concentration (5.04 g/L) is obtained when the initial fructose concentration of the medium is 98 g/L and the temperature is 27.9 °C.

It has been determined that the maximum liamocin concentration is higher than 5 g/L under conditions where the initial fructose concentration ranged between 93 and 102 g/L and the temperature ranged between 27 and 28 °C. The maximum liamocin concentration was quite low (<2 g/L) when the initial fructose concentration was at the highest values in the studied range (127–129 g/L) and the temperature was at the lowest values within the range of 25–26 °C (Figure 1b).

In Figure 1c, response surface and contour graphs showing the effects of the initial pH (*X*2) and temperature values (*X*3) of the fermentation medium on the maximum liamocin concentration are presented. As can be seen from the response surface plot in Figure 2c, in the fermentation medium with an initial fructose concentration of 100 g/L, the liamocin concentration ranged between 1.9 and 2.5 g/L at high temperature (30 °C) and low pH values (3.8–4.0). High liamocin concentrations were reached in conditions where the temperature varied between 26 and 29 °C and the initial pH of the medium varied between 4.3 and 6.2. In this range, it was determined that the maximum liamocin concentration varied between 4.5 and 5.08 g/L.

**Figure 2:**
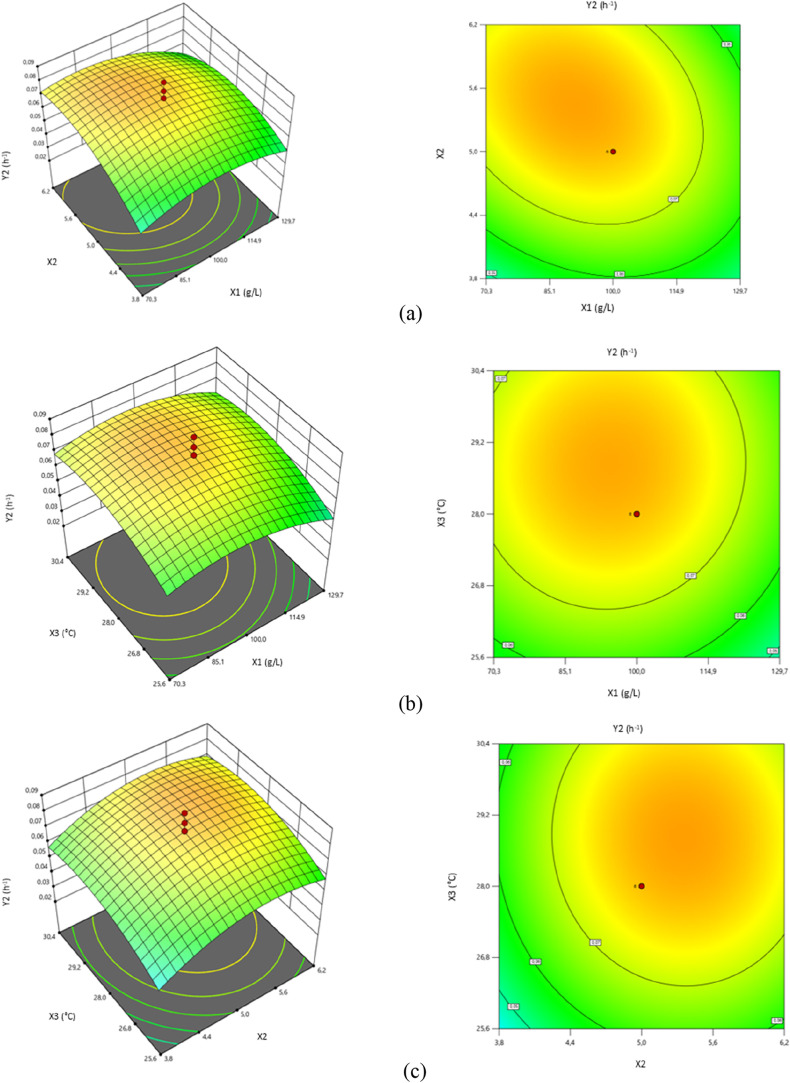
Response surface and contour graphs of microorganism-specific growth rates (*Y*2) as a function of (a) initial fructose concentration (*X*1) and initial pH (*X*2), (b) initial fructose concentration (*X*1) and temperature (*X*3), and (c) initial pH (*X*2) and temperature (*X*3).

According to the contour plot in Figure 1c, the liamocin concentration was determined to be above 5 g/L at temperatures of 27–28 °C and pH of 4.9–5.8. It can be seen that the maximum liamocin concentration was 5.1 g/L when the initial fructose concentration, pH, and temperature were at 100 g/L, 5.29, and 27.7 °C, respectively. At lower values of the studied temperature and pH ranges, the maximum liamocin concentration decreased. The maximum liamocin concentration was determined to be 2.90 g/L when the temperature was 25.8 °C and initial pH was 3.84.

The quadratic model equation predicted for the effects of initial fructose concentration (*X*1), initial pH (*X*2), and temperature (*X*3) on the microorganism-specific growth rate (*Y*2) is shown below

8 $$Y2=+0.0775-0.0039X1+0.0067X2+0.0061X3-0.0053X1X2+0.0005X1X3-0.0005X2X3-0.0099{\left(X1\right)}^{2}-0.0113{\left(X2\right)}^{2}-0.0088{\left(X3\right)}^{2}$$

The coefficient of determination for the derived model equation is calculated as *R* ^2^ = 0.7905. Table 4 presents the results of the variance analysis of the model equation.

**Table 4:** ANOVA Results for the Quadratic Model Predicted for the Microorganism-Specific Growth Rate (*Y*2)

source	df	mean squares	*F*-value	*p*-value	remarks
model	9	6.00 × 10^–4^	4.19^a^	0.0177	significant
*X*1	1	2.00 × 10^–4^	1.51	0.2467	not significant
*X*2	1	6.00 × 10^–4^	4.43	0.0615	not significant
*X*3	1	5.00 × 10^–4^	3.71	0.0830	not significant
*X*1*X*2	1	2.00 × 10^–4^	1.59	0.2359	not significant
*X*1*X*3	1	2.00 × 10^–6^	0.0144	0.9068	not significant
*X*2*X*3	1	2.00 × 10^–6^	0.0144	0.9068	not significant
*X*1^2^	1	1.40 × 10^–3^	10.19^a^	0.0096	significant
*X*2^2^	1	1.80 × 10^–3^	13.30^a^	0.0045	significant
*X*3^2^	1	1.10 × 10^–3^	8.12^a^	0.0173	significant
lack of fit	5	2.00 × 10^–4^	2.42	0.1770	not significant
pure error	5	1.00 × 10^–4^			
cor total	19				

a *p* < 0.05.

The model *F*-value of 4.19 implies the model is significant. There is only a 1.77% chance that an *F*-value this large could occur due to noise. And the “lack of fit *F*-value” of 2.42 implies the lack of fit is not significant relative to the pure error. There is a 17.70% chance that a “lack of fit *F*-value” this large could occur due to noise.

The results presented in Table 2 show that the model is significant (*p* < 0.05), and the lack of fit of the derived model is insignificant at the 95% confidence level (*p* > 0.05). It was found that the binary interactions of all independent variables examined on the microorganism-specific growth rate (*Y*2) were insignificant (*p* > 0.05) and their quadratic effects were significant (*p* < 0.05).

The three-dimensional (3D) response surface and contour graphs showing the effects of the initial fructose concentration (*X*1), initial pH (*X*2), and temperature (*X*3) of the fermentation medium on the specific growth rate (*Y*2) of the *A. pullulans* NBRC 100716 strain are shown in Figure 2.

According to Figure 2a, microorganism-specific growth rates (μ) were also determined to be low at low fructose initial concentration and pH values in the studied range. In the condition that the initial concentration of fructose was 71 g/L and the initial pH was 3.8, the value of μ was determined to be 0.049 h^–1^. The specific growth rate was determined to be over 0.070 h^–1^ under conditions where initial fructose concentrations ranged between 83 and 112 g/L and initial pH ranged between 4.3 and 6.2. As can be seen from the graphs in Figure 2a, the highest microorganism-specific growth rate (0.079 h^–1^) was obtained when the initial fructose concentration was 90.6 g/L and the initial pH was 5.4 (Figure 2a).

Graphs showing the effect of the fructose initial concentration (*X*1) and temperature (*X*3) of the fermentation medium on the specific growth rate of microorganisms (*Y*2) are given in Figure 2b. According to the response surface graph, it was determined that the microorganism-specific growth rate was at the minimum value (0.049 h^–1^) under conditions where the initial fructose concentration was 129.3 g/L and the temperature was 25.6 °C. With the increase in the initial fructose concentration when the temperature was between 25.6 and 26.5 °C, the specific growth rate values (*Y*2) of *A. pullulans* NBRC 100716 were also increased. High *Y*2 values were achieved under conditions where the temperature varied between 26.4 and 30.4 °C and the initial fructose concentration ranged between 75 and 112 g/L. The specific growth rate was found to be 0.078 h^–1^ when the initial fructose concentration was 99.2 g/L and the temperature was 28.4 °C.

Response surface and contour graphs showing the effects of the initial pH (*X*2) and temperature (*X*3) of the fermentation medium on the specific growth rate of the microorganism (*Y*2; μ) are presented in Figure 2c. As could be seen in the response surface graph, it was determined that the microorganism-specific growth rate was quite low when the initial pH of the fermentation medium ranged from 3.8 and 4.05, and the temperature was between 25.6 and 26.2 °C. When the pH is 3.8 and the temperature is 25.6 °C, the microorganism-specific growth rate is 0.044 h^–1^. High specific growth rate values were reached when the initial pH was in the range of 4.30–6.15 and the temperature was changed from 26.5 to 30.3 °C. Microorganism-specific growth rates over 0.070 h^–1^ were achieved at values of *X*2 and *X*3 within the specified ranges. The microorganism-specific growth rate was determined as 0.079 h^–1^ when the initial pH and temperature of the fermentation medium were 5.36 and 28.5 °C, respectively.

When the contour graph in Figure 2c was examined, it was determined that the specific growth rate was lower than 0.05 h^–1^ for low pH (3.8–4.0) and temperature values (25.6–26.1 °C) in the studied range. It has been determined that under conditions where the temperature ranged between 28.8 and 30.4 °C and the initial pH ranged between 4.6 and 6.0, specific growth rate values are high and changed between 0.076 and 0.079 h^–1^. According to the contour plot, when the temperature is 29.5 °C and the pH is 4.90, *Y*2 is obtained as 0.077 h^–1^.

The model equation showing the effects of initial fructose concentration (*X*1), initial pH (*X*2), and temperature (*X*3) on the maximum specific liamocin production rate (*Y*3) is presented below

9 $$Y3=+0.0060-0.0001X1+0.0005X2+0.000X3-0.0002X2X3-0.0017{\left(X1\right)}^{2}-0.0008{\left(X2\right)}^{2}-0.0014{\left(X3\right)}^{2}$$

For maximum specific liamocin production rate values, the *R* ^2^ value of the model is 0.8512. The fact that this value is close to 1 states that experimental *Y*3 values and *Y*3 values calculated from eq 9 are close to each other. ANOVA results of this model are presented in Table 5.

**Table 5:** ANOVA Results for the Quadratic Model Predicted for the Maximum Specific Liamocin Production Rate (*Y*3)

source	df	mean squares	*F*-value	*p*-value	remarks
model	9	8.153 × 10^–6^	6.36^a^	0.0039	significant
*X*1	1	2.758 × 10^–7^	0.2150	0.6528	not significant
*X*2	1	3.801 × 10^–6^	2.96	0.1159	not significant
*X*3	1	1.618 × 10^–7^	0.1261	0.7299	not significant
*X*1*X*2	1	1.250 × 10^–9^	0.0010	0.9757	not significant
*X*1*X*3	1	1.250 × 10^–9^	0.0010	0.9757	not significant
*X*2*X*3	1	3.612 × 10^–7^	0.2816	0.6072	not significant
*X*1^2^	1	0.0000	32.57^a^	0.0002	significant
*X*2^2^	1	9.662 × 10^–6^	7.53^a^	0.0207	significant
*X*3^2^	1	0.0000	22.65^a^	0.0008	significant
lack of fit	5	1.839 × 10^–6^	2.53	0.1656	not significant
pure error	5	7.267 × 10^–7^			
cor total	19				

a *p* < 0.05.

The model *F*-value of 6.36 implies the model is significant. There is only a 0.39% chance that an *F*-value this large could occur due to noise. The “lack of fit *F*-value” of 2.53 implies that lack of fit is not significant relative to the pure error. There is a 16.56% chance that a “lack of fit *F*-value” this large could occur due to noise.

According to the variance analysis results of the model, it was determined that the model was significant (*p* < 0.05) and the lack of fit of the model was insignificant (*p* > 0.05) at the 95% confidence level. The quadratic effects of all independent variables were found to be significant (*p* < 0.05) for the maximum specific liamocin production rate (*Y*3) (*p* < 0.05).

3D response surface and contour graphs showing the effects of the two independent variables examined in this study on the maximum specific liamocin production rate (*Y*3) are presented in Figure 3.

**Figure 3:**
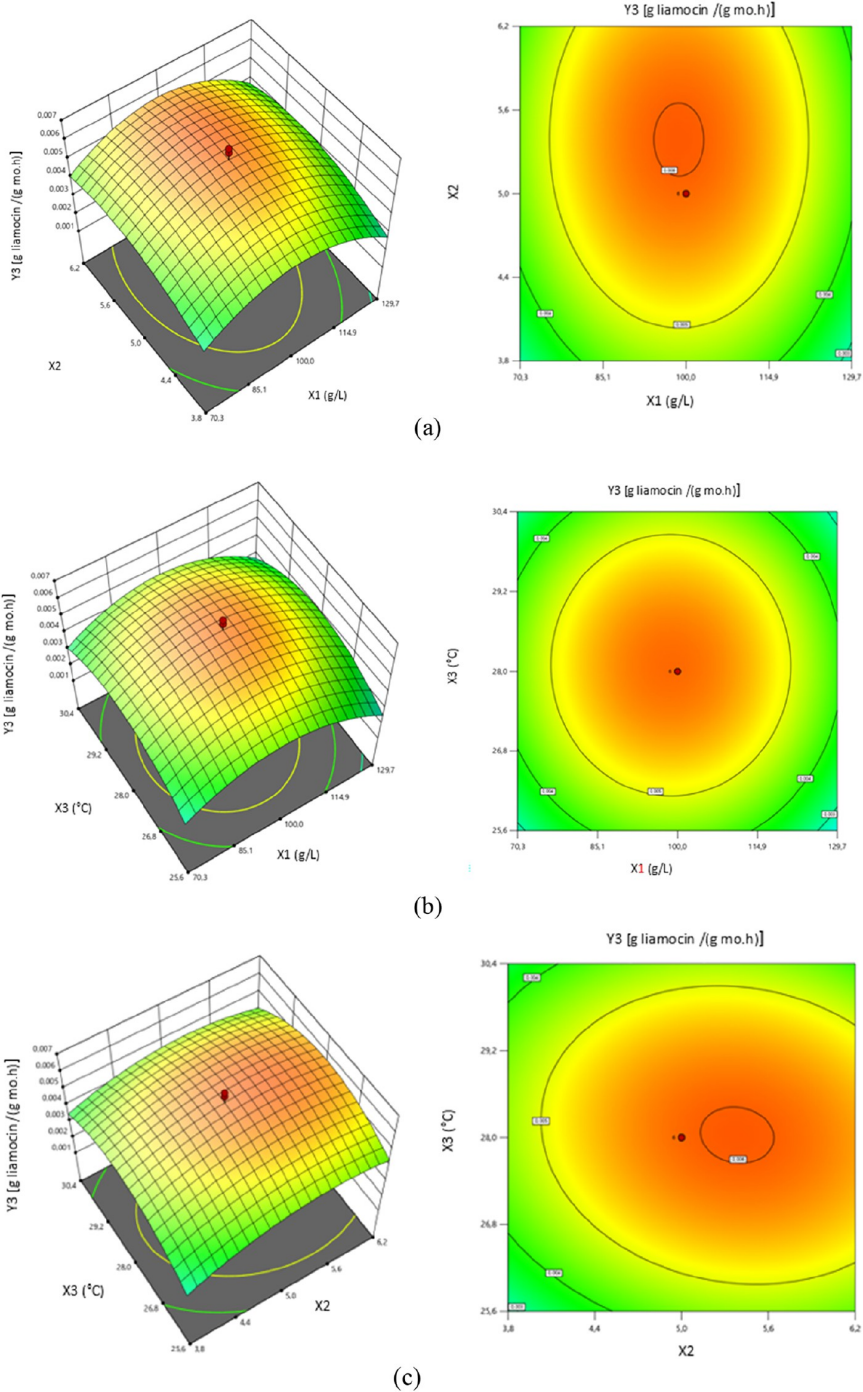
Response surface and contour graph of the maximum specific liamocin production rate (*Y*3) as a function of (a) initial fructose concentration (*X*1) and initial pH (*X*2), (b) initial fructose concentration (*X*1) and temperature (*X*3), and (c) initial pH (*X*2) and temperature (*X*3).

At pH 3.8, the maximum specific liamocin production rates were determined to be low at the lowest and highest fructose initial concentrations in the studied range (Figure 3a). The highest *Y*3 values were obtained when the independent variables were close to their central values.

According to the contour plot indicating interactions of *Y*3 with *X*1 and *X*3, under the condition that pH was 3.82 and fructose initial concentration was 129.4 g/L, *Y*3 was determined as 0.0028 [g liamocin/(g mo·h)]. It was determined that the *Y*3 values were above 0.006 [g of liamocin/(g mo·h)] under conditions where the initial fructose concentration ranged between 94.3 and 103.0 g/L and the initial pH ranged between 5.12 and 5.62 (Figure 3b).

Figure 3b presents response surface and contour graphs showing the effects of the fermentation medium initial fructose concentration (*X*1) and temperature (*X*3) on the maximum specific liamocin production rate (*Y*3). As can be seen from the 3D response surface graph, for low *X*1 and *X*3 values in the studied range, the *Y*3 values were also found to be low. In the same graph, the maximum specific liamocin production rate was found to be 0.0029 [g liamocin/(g mo·h)] when the initial fructose concentration was 70.74 g/L and the temperature was 25.7 °C. It was determined that high *Y*3 values were achieved under conditions where the initial fructose concentration was between 85.6 and 116.0 g/L and the temperature ranged between 26.8 and 29.0 °C. In these specified ranges, the maximum specific liamocin production rates are higher than 0.0055 [g liamocin/(g mo·h)]. When the initial fructose concentration was 99.6 g/L and the temperature was 28.2 °C, *Y*3 was determined as 0.0059 [g liamocin/(g mo·h)] (Figure 3b).

Figure 3c shows the effects of the fermentation medium initial pH (*X*2) and temperature (*X*3) values on the maximum liamocin production rate (*Y*3). As seen in the response surface graph given in Figure 3c, high *Y*3 values were obtained when the initial pH was in the range between 4.42 and 6.17 and the temperature was in the range between 26.5 and 29.7 °C. According to the contour graph in Figure 3c, the maximum specific liamocin production rates were low at low pH and temperature values in the studied ranges. It was determined that the *Y*3 was above 0.006 [g liamocin/(g mo·h)] when the initial pH of the fermentation medium ranged between 5.12 and 5.62 and temperature was between 27.6 and 28.4 °C.

### 3.2 Optimization and Validation of Liamocin Production

The optimum conditions used in this study were selected from 58 solutions produced by the program used (see Section 2.10) with the desirability functions from 0.814 to 0.825.

The conditions of the solution, whose desirability function was chosen as 0.825, the value closest to 1, were the initial concentration of fructose in the fermentation medium was 93.47 g/L, the initial pH was 4.92, and the temperature was 27.2 °C. Under these conditions, the maximum liamocin concentration, microorganism-specific production rate, and maximum specific liamocin production rate were estimated to be 4.90 g/L, 0.07447 h^–1^, and 0.00567 [g liamocin/(g mo·h)], respectively. After determining the operating conditions under which the optimization process would be performed, an experiment was carried out under optimum conditions to determine the accuracy of the estimated values. Experiments under optimum conditions were carried out as described in Section 2.10. The initial inoculation concentration of the tested strain was determined as 6.8 × 10^7^ CFU/mL for these experiments.

Figure 4 shows the changes in liamocin, biomass (*X*), and fructose concentrations, and substrate consumption rates (*r_S_*) with time in optimum operating conditions.

**Figure 4:**
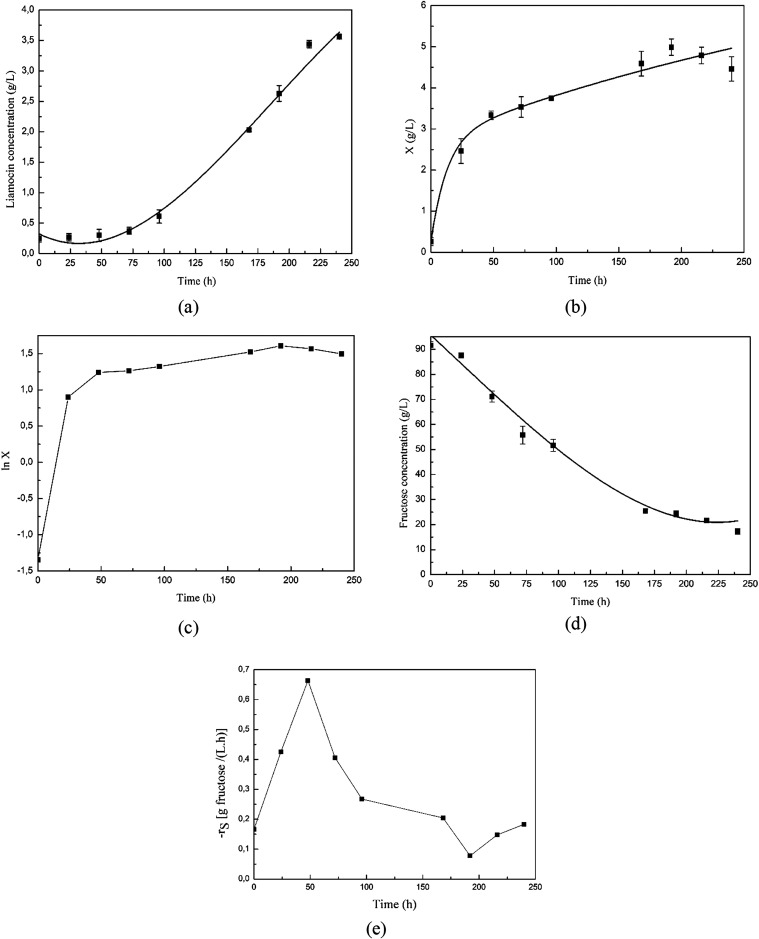
Variations of (a) liamocin, (b) biomass (*X*), (c) ln *X*, (d) fructose concentrations, and (e) fructose consumption rate (*r_S_*) with time in the optimum operating conditions.

The highest liamocin concentration obtained by *A. pullulans* NBRC 100716 strain was determined as 3.56 ± 0.04 g/L for the experiment performed in optimum conditions (Figure 4a).

The graph showing the change in biomass concentration with time is shown in Figure 4b. It was found that under optimum fermentation conditions, the biomass concentration increased with time and the highest biomass concentration was 4.99 ± 0.20 g/L.

The specific microbial growth rate (μ) was calculated using the biomass concentration results of the *A. pullulans* NBRC 100716 strain. Equation 1 (Section 2.7) was used for this calculation. For this purpose, the μ value in the exponential phase was calculated by drawing a graph showing the changes in ln *X* values with time (Figure 4c). The specific growth rate of the tested strain under optimum fermentation conditions was calculated as 0.067 h^–1^.

The maximum specific liamocin production rate (qp) of the *A. pullulans* NBRC 100716 strain was calculated as 0.0045 g liamocin/(g mo·h) for the experiments carried out under optimum conditions. The graphs given in Figure 4a,4b were used to calculate the qp value.

Figure 4d presents the graph showing the change in the fructose concentration in the fermentation medium with time. For this experiment, it was determined that the fructose concentration in the medium decreased with time and some fructose (17.30 ± 1.17 g/L) remained in the medium at the end of fermentation. The graph showing the change in the fructose consumption rate (*r_S_*) with time is presented in Figure 4e. The maximum fructose consumption rate for these conditions was calculated as 0.66 g fructose/(L·h) at the 48th hour of fermentation.

The experimental values of the dependent variables obtained as a result of this study and the theoretical values calculated using eqs 7–9 are presented in Table 6.

**Table 6:** Experimental Values of Dependent Variables and Their Theoretical, Minimum, and Maximum Values Calculated Using the Optimization Program^a^

dependent variable	experimental value	theoretical value	minimum value	maximum value
*Y*1	3.56	4.90	1.80	8.00
*Y*2	0.0670	0.0744	0.04619	0.1027
*Y*3	0.0045	0.0057	0.00295	0.0084

a *Y*1: Maximum liamocin concentration (g/L), *Y*2: microorganism-specific growth rate (h^–1^), *Y*3: maximum specific liamocin production rate [g liamocin/(g mo·h)].

After the postanalysis conducted by the program, it was predicted that maximum liamocin concentration should be 1.80 and 8.00 g/L in 95% low and high confidence levels, respectively. Similarly, microorganism-specific growth rates were expected to be 0.046 and 0.103 h^–1^ and maximum specific liamocin production rates were expected to be 0.003 and 0.008 g liamocin/(g mo·h) in 95% confidence interval.

As seen in Table 6, the experimental values of the dependent variables are very close to the theoretical values, and these results are between the minimum and maximum values calculated with the help of the relevant program. The experimental values were confirmed to be in 95% confidence interval.

Researches on the optimization of various operational conditions in liamocin production by fermentation are limited. In the study conducted by Leathers et al.,^11^ for the optimization of medium components for the liamocin production of *A. pullulans* NRRL 50384 strain in the batch fermentation system, Placket Burman design, one-factor-at-a-time method was used. In the mentioned study, it was stated that an experimental design was carried out by selecting the highest and lowest values for each experimental variable, but it was reported that none of the model terms were statistically significant (*p* > 0.05) among the values tested as a result of ANOVA. As a result, it was reported that the liamocin concentration (22 g/L) produced by *A. pullulans* NRRL 50384 strain in the optimized medium increased approximately 2-fold compared to the standard medium.

In the most recent study in the literature on this subject, a minimal medium was designed and optimized to enhance the economic efficiency of the process and improve the industrial applicability of liamocin production.^12^ In the study mentioned above, the polyol lipid concentration obtained with *A. pullulans* NRRL 62042 strain in the optimized medium was reported as 48 g/L.

In a study conducted by Manitchotpisit et al.,^32^ liamocin production of different *A. pullulans* strains was investigated in a batch system and it was reported that liamocin concentrations produced by 9 different strains varied between 0.3 and 8.6 g/L. *A. pullulans* NRRL 62042 strain was determined as the best liamocin producer (8.6 g/L).

Our findings for the maximum liamocin production (Table 2, 6.10 g/L) can be evaluated as satisfactory compared to the results obtained in different studies.

In addition, although there are few studies on the optimization of medium composition in the production of liamocin with different *A. pullulans* strains, to the best of our knowledge, there are no studies on the optimization of some critical fermentation parameters, such as pH, temperature, and initial carbon source concentration of the fermentation medium. In this sense, our research is the first to optimize various fermentation parameters in liamocin production with the *A. pullulans* NBRC 100716 strain.

### 3.3 Results of the Characterization of Liamocin Samples

#### 3.3.1 Results of Nile Red Staining

Fluorescence microscope images obtained after the Nile red staining method was applied to the slides prepared from the *A. pullulans* culture used in the experiments are given in Figure 5. Fluorescent oil droplets inside and outside the cells were visualized in bright red, an indicator of oil (liamocin) production of the studied strain.

**Figure 5:**
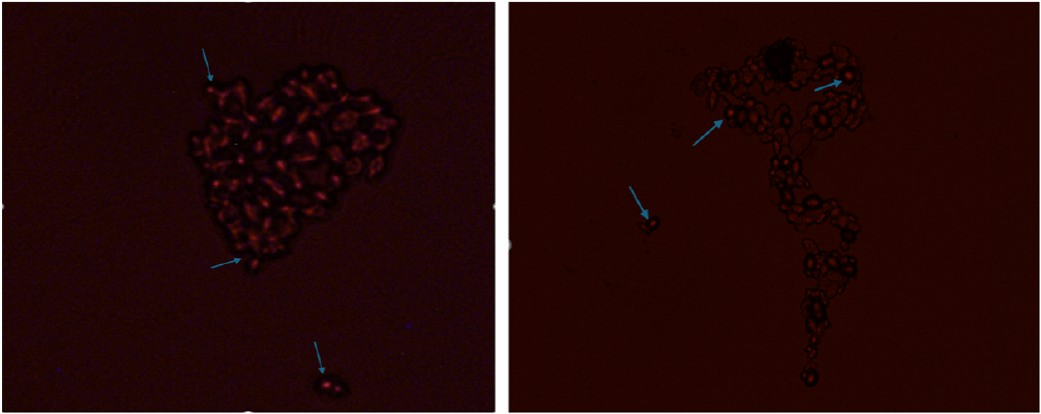
Fluorescence microscope images of *A. pullulans* NBRC 100716 cells stained with Nile red (Nikon Eclipse TS100, 40×).

#### 3.3.2 Results of Oil Red O staining

As can be seen in the images (Figure 6), intracellular and extracellular lipid droplets are stained red. During staining, Oil red O is transferred from the dye solution to the lipid, resulting in the lipid droplets being stained red.^27^ With this staining method used, the production of liamocin, also defined as heavy oil, of the tested strain was confirmed. Additionally, to the best of our knowledge, this is the first study to visually present liamocin droplets by staining them with Oil red O.

**Figure 6:**
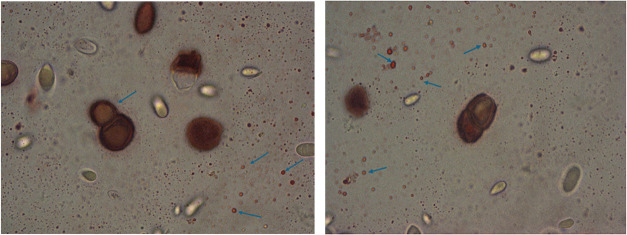
Results of Oil red O staining of *A. pullulans* NBRC 100716 culture (100× magnification).

#### 3.3.3 Results of Structural Characterization

The molecular structure of the liamocin sample produced by the *A. pullulans* NBRC 100716 strain was analyzed by MALDI-TOF-MS and the obtained spectrum is presented in Figure 7.

**Figure 7:**
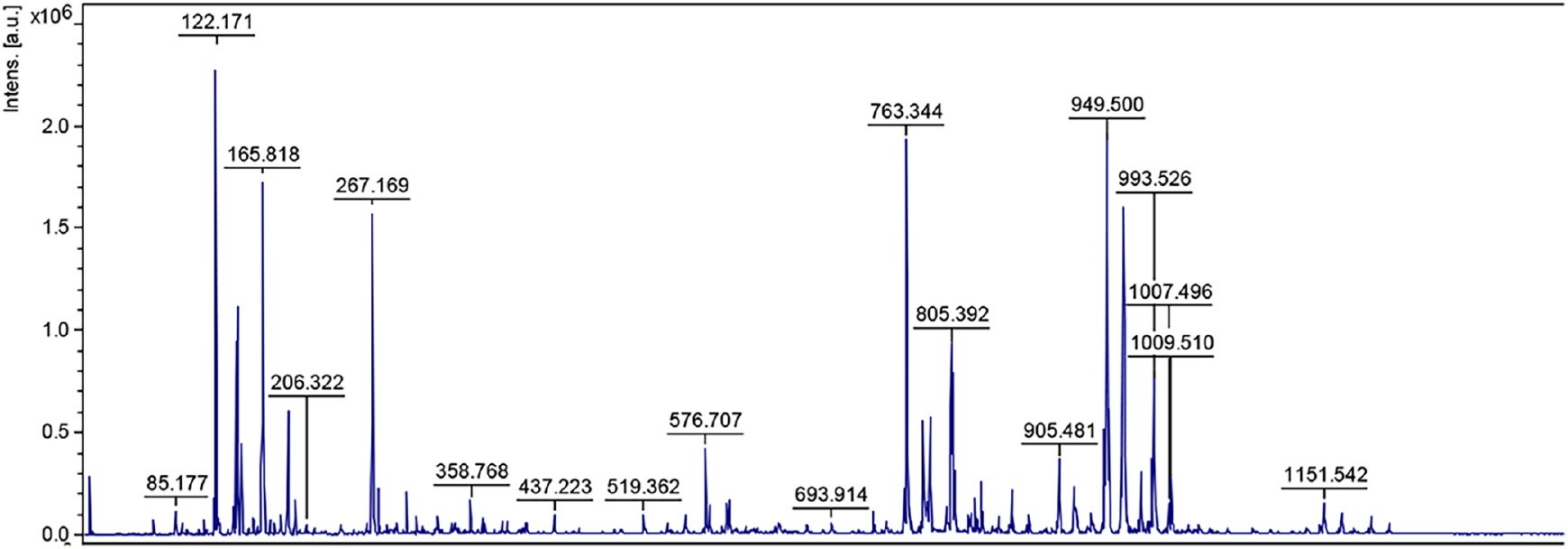
MALDI-TOF-MS spectrum of the liamocin sample.

The analysis was performed in positive ion mode, resulting in pseudomolecular [M + Na]^+^ adduct ions within the mass range of 750–1010 Da. It was confirmed that the liamocin produced by the tested strain includes liamocin A1, B1, A2, and B2 and corresponds to the molecular structure given in the literature.

Liamocin produced by the NBRC 100716 strain predominantly contains liamocin B1 (*m*/*z* 949.5) and liamocin A1 molecules (*m*/*z* 763.3) (Figure 7). The relative abundance of liamocin A1, A2, B1, and B2 were determined as 33.02, 15.60, 36.54, and 14.85%, respectively. According to the previous reports, peaks at *m*/*z* 763.4 and 949.5 correspond to nonacetylated liamocins, whereas the peak at *m*/*z* 805.4 is associated with monoacetylated compounds.

In the literature, it is stated that the *m*/*z* 763.4 and *m*/*z* 949.5 peaks are caused by the nonacetylated liamocin compound and the peak at 805.4 is caused by monoacetylated compounds.^29^ In our study, the peaks obtained in the MALDI-TOF-MS spectrum were found to be compatible with the peaks given in the literature. The spectrum indicates that there are two types of nonacetylated liamocin (Liamocin A1 and B1) with masses of 740.2 and 926.4 Da giving peaks to [M + Na]^+^ *m*/*z* 763.2 and 949.4 ions, respectively. Similarly, there are two types of monoacetylated liamocin A2 and B2 with masses of 782.2 and 968.4 Da giving 805.2 and 991.4 peaks.

#### 3.3.4 Results of GC-FID Analysis

The fatty acid composition of the analyzed liamocin sample is shown in Table 7.

**Table 7:** Fatty Acid Composition of the Liamocin Sample Produced by *A. pullulans* NBRC 100716 Strain^a^

retention time (min)	fatty acid	relative area (%)
4.69	caproic acid (C_6:0_)	0.02
8.15	caprylic acid (C_8:0_)	0.01
12.51	capric acid (C_10:0_)	0.04
14.89	undecanoic acid (C_11:0_)	0.04
17.14	lauric acid (C_12:0_)	0.01
19.89	tridecanoic acid (C_13:0_)	0.06
22.70	myristic acid (C_14:0_)	0.09
25.77	pentadecanoic acid (C_15:0_)	0.03
29.12	palmitic acid (C_16:0_)	25.00
30.55	palmitoleic acid (C_16:1_)	0.45
32.14	heptadecanoic acid (C_17:0_)	0.15
35.67	stearic acid (C_18:0_)	34.49
36.9	oleic acid (C_18:1_)	33.45
39.07	linoleic acid (C_18:2_)	4.80
41.56	arachidic acid (C_20:0_)	0.92
41.82	α-linolenic acid (C_18:3_)	0.10
42.77	eicosenoic acid (C_20:1_)	0.07
47.76	behenic acid (C_22:0_)	0.14
53.12	lignoceric acid (C_24:0_)	0.12

a As an external standard, Supelco 37 Component FAME Mix (Sigma-Aldrich) was used (see Section 2.11.4).

It was determined that the fatty acid composition of the liamocin sample produced by *A. pullulans* NBRC 100716 strain consisted predominantly of palmitic (C16:0; 25.00%), stearic (C18:0; 34.49%), oleic (C18:1; 33.45%), and α-linoleic (C18:2; 4.80%) acids.

The major fatty acids produced by oleaginous yeasts are thought to be considered to be similar to those produced by plants and consist mainly of myristic (C14:0), palmitic (C16:0), palmitoleic (C16:1), stearic (C18:0), oleic (C18:1), linoleic (C18:2) and linolenic (C18:3) acids.^33^

In a study investigating single-cell oil production by *Aureobasidium pullulans* var. *melanogenum* P10 strain, the fatty acid composition of the produced oil was determined.^30^ In the aforementioned study, the fatty acid composition was reported mainly as palmitic acid (C16:0; 26.7%), palmitoleic acid (C16:1; 1.7%), stearic acid (C18:0; 6.1%), oleic acid (C18:1; 44.5%), and linoleic acid (C18:2; 21%).

It was determined that the *A. pullulans* NBRC 100716 strain used in this study produced heavy oil (liamocin) with a fatty acid composition similar to that of vegetable oils, which is compatible with studies on single-cell oil in the literature.

Liamocin produced by *A. pullulans* NBRC 100716 strain has a high content of saturated fatty acids (SFA) (61.13%), and a significant amount of unsaturated fatty acids (UFA) (38.87%). Monounsaturated fatty acid (MUFA) and polyunsaturated fatty acid (PUFA) ratios in the UFA were 33.97 and 4.90%, respectively.

Determining the fatty acid profiles of lipids produced by yeasts is important in terms of potential usage areas. Lipids rich in monounsaturated fatty acids (MUFA) are preferred for biodiesel production due to their oxidative stability. Additionally, they play a key role in the pharmaceutical and nutrition industries since they contribute to cardiovascular health by lowering LDL cholesterol in human nutrition. Lipids rich in polyunsaturated fatty acids (PUFA) are more valuable for animal feed and human food as they provide essential fatty acids that cannot be synthesized by the human body and must be obtained from the diet.^34,35^

MUFA and PUFA amounts of liamocin produced in this study were determined as 33.97 and 4.90%, respectively. In this respect, the liamocin we obtained in our research was found to be promising as it could be a potential sustainable source for biodiesel production, pharmaceutical, and nutrition industries due to its high unsaturated fatty acid content.

#### 3.3.5 Results of FT-IR Analysis

The FT-IR spectrum of the liamocin sample produced by the *A. pullulans* NBRC 100716 strain is given in Figure 8.

**Figure 8:**
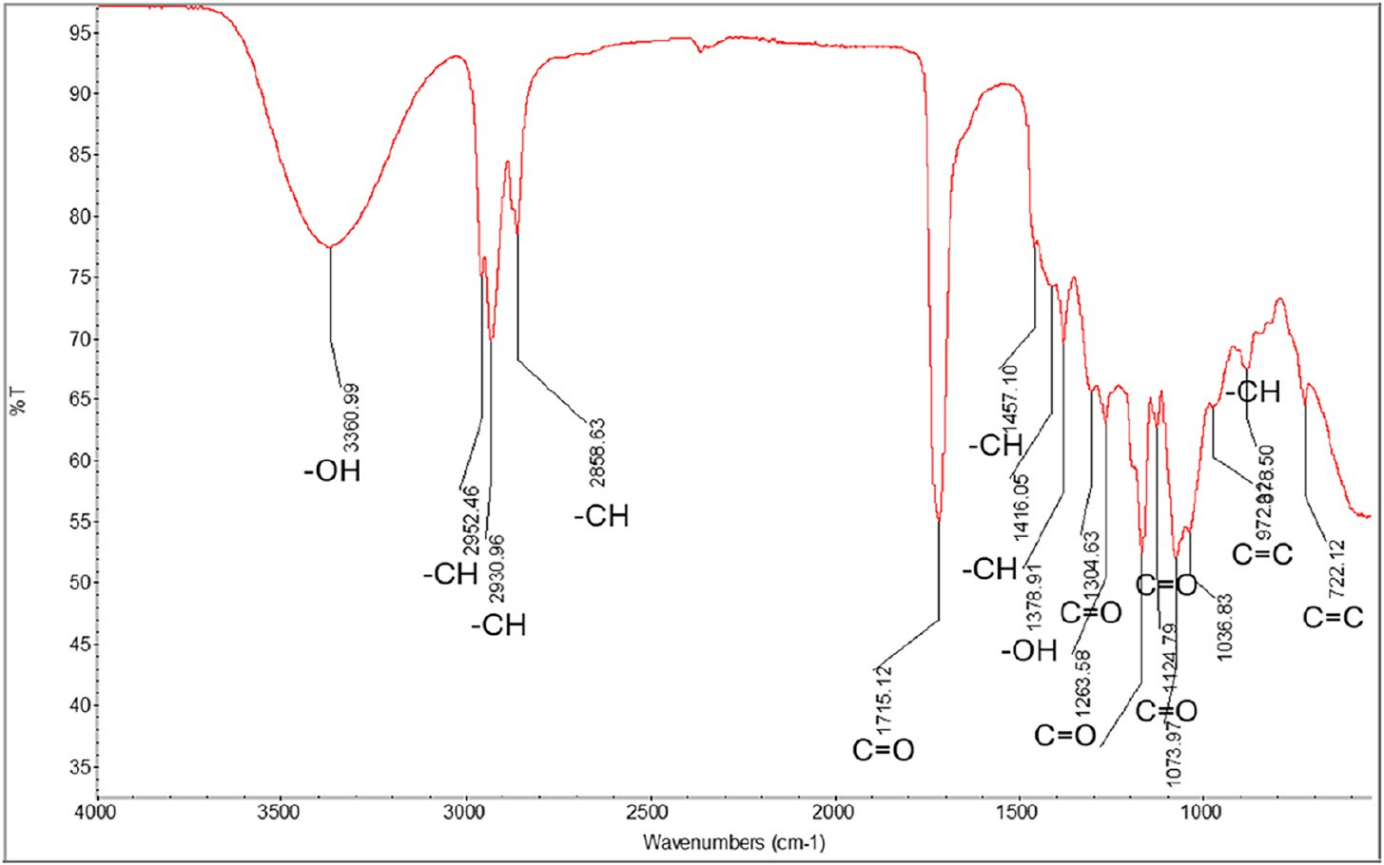
FT-IR spectrum of the liamocin sample.

As can be seen in Figure 8, O–H stretching is at 3360 cm^–1^, the C–H peaks at 2952.46, 2930.96, and 2858.63 cm^–1^. The = C–H corresponding to the fatty acid peaks at 2952 cm^–1^. In the FT-IR analysis of the biosurfactant obtained in a study on the production of eco-friendly biosurfactant, the presence of long aliphatic chains (CH, CH_2_, CH_3_) was clearly shown by the absorption peaks between 2930 and 2858 cm^–1^, and the absorption peaks at 1457 and 1416 cm^–1^ were associated with the C–H alkyl bond vibration.^36^

The C=O stretching peak at 1715.12 cm^–1^ (Figure 8) can be observed. It is thought that the peaks between 1073.97 and 1304.63 cm^–1^ are caused by C–O stretching. While the peaks at 722.12 and 972.00 cm^–1^ were caused by C=C bending, it was determined that C–H bending was responsible for the peak at 878.50 cm^–1^.

The carbonyl group of ester has also appeared at 1304 cm^–1^, which corresponds to the vibrations of C–O, proving that almost all biosurfactants have an ester group or carboxylic acid group of their structure.^37,38^

The adsorption bands located at 1378 or 1457 cm^–1^ in the spectrum given in Figure 8 show that the molecule has a chemical structure identical to that of the glycolipids. The band at 1378 cm^–1^ represents the −OH group and the band at 1457 cm^–1^ represents the −CH group, indicating that liamocin has both hydrophilic and hydrophobic components, which are characteristic of surface-active molecules.^38^

In our previous study, it was determined that liamocin produced by the *A. pullulans* NBRC 100716 strain has surface-active character. The liamocin sample reduced the surface tension of water from 72.30 to 31.60 mN/m and the critical micelle concentration was calculated as 0.004 mg/mL.^9^

Since there is no previous study that revealed the structure of liamocin by FT-IR analysis, comparison with previous data could not be made. However, the FT-IR spectrum obtained for liamocin in this study was evaluated with the results of studies on glycolipid-type biosurfactants in the literature.^36,38^ As a result, findings were obtained, indicating that the produced liamocin sample had a structure similar to that of the studies mentioned above.

## 4 Conclusions

This study makes a significant contribution to the improvement of fermentation conditions for liamocin production, with the *A. pullulans* NBRC 1007165 strain used for the first time. The use of response surface methodology (RSM) enabled the identification of optimum conditions for liamocin production. In optimized fermentation conditions, the maximum liamocin concentration, microorganism-specific growth rate, and maximum specific liamocin production rate were determined as 3.56 g/L, 0.0670 h^–1^, and 0.00450 [g liamocin/(g mo·h)], respectively.

The fatty acid composition of liamocin, whose molecular structure was confirmed by MALDI-TOF-MS, was determined for the first time in this study. The similarity of the fatty acid profile of liamocin produced by the *A. pullulans* NBRC 100716 strain to that of vegetable oils was found to be promising for future studies.

FT-IR analysis of liamocin, whose surface-active character was revealed in our previous study, gave a spectrum indicating that the molecule is a glycolipid-type biosurfactant containing hydrophilic and hydrophobic groups. To the best of our knowledge, this is also the first report in which the molecular structure of liamocin was determined by FT-IR.

Optimization of fermentation conditions and study of fermentation kinetics are of great importance for industrial-scale production of liamocins, and such production still appears to be economically costly. In our further studies, it is planned to use some agricultural wastes containing significant amounts of organic and inorganic resources for the production of liamocin, thus, reducing large-scale production costs. In addition, future studies are aimed to better understand the potential biotechnological applications of liamocin, including the food sector, by conducting more studies on the production of glycolipid-type biosurfactants produced by *A. pullulans* NBRC 100716 strain.

This study revealed that the *A. pullulans* NBRC 100716 strain used in the experiments is one of the rare strains that produces promising amounts of liamocin without genetic manipulation and has the potential for large-scale production with optimization of some fermentation conditions.
